# Towards unravelling biological mechanisms behind radiation-induced oral mucositis via mass spectrometry-based proteomics

**DOI:** 10.3389/fonc.2023.1180642

**Published:** 2023-06-13

**Authors:** Prabal Subedi, Katharina Huber, Christoph Sterr, Anne Dietz, Lukas Strasser, Felix Kaestle, Stefanie M. Hauck, Lukas Duchrow, Christine Aldrian, Elsa Beatriz Monroy Ordonez, Benedikt Luka, Andreas R. Thomsen, Michael Henke, Maria Gomolka, Ute Rößler, Omid Azimzadeh, Simone Moertl, Sabine Hornhardt

**Affiliations:** ^1^ Bundesamt für Strahlenschutz/Federal Office for Radiation Protection, Neuherberg, Germany; ^2^ Helmholtz Zentrum München, German Research Centre for Environmental Health, Metabolomics and Proteomics Core, Munich, Germany; ^3^ Department of Radiation Oncology, Medical Center, Faculty of Medicine, University of Freiburg, German Cancer Consortium (DKTK) partner site Freiburg, Freiburg, Germany; ^4^ Department of Conservative Dentistry Periodontology and Preventive Dentistry, Hannover Medical School (MHH), Hannover, Germany; ^5^ German Cancer Consortium (DKTK) Partner Site Freiburg, German Cancer Research Center (dkfz), Heidelberg, Germany

**Keywords:** radiotherapy, keratinocytes, biomarker, mass spectrometry-based proteomics, interferon response, STAT phosphorylation, MCM complex

## Abstract

**Objective:**

Head and neck cancer (HNC) accounts for almost 890,000 new cases per year. Radiotherapy (RT) is used to treat the majority of these patients. A common side-effect of RT is the onset of oral mucositis, which decreases the quality of life and represents the major dose-limiting factor in RT. To understand the origin of oral mucositis, the biological mechanisms post-ionizing radiation (IR) need to be clarified. Such knowledge is valuable to develop new treatment targets for oral mucositis and markers for the early identification of “at-risk” patients.

**Methods:**

Primary keratinocytes from healthy volunteers were biopsied, irradiated *in vitro* (0 and 6 Gy), and subjected to mass spectrometry-based analyses 96 h after irradiation. Web-based tools were used to predict triggered biological pathways. The results were validated in the OKF6 cell culture model. Immunoblotting and mRNA validation was performed and cytokines present in cell culture media post-IR were quantified.

**Results:**

Mass spectrometry-based proteomics identified 5879 proteins in primary keratinocytes and 4597 proteins in OKF6 cells. Amongst them, 212 proteins in primary keratinocytes and 169 proteins in OKF6 cells were differentially abundant 96 h after 6 Gy irradiation compared to sham-irradiated controls. *In silico* pathway enrichment analysis predicted interferon (IFN) response and DNA strand elongation pathways as mostly affected pathways in both cell systems. Immunoblot validations showed a decrease in minichromosome maintenance (MCM) complex proteins 2-7 and an increase in IFN-associated proteins STAT1 and ISG15. In line with affected IFN signalling, mRNA levels of IFNβ and interleukin 6 (IL-6) increased significantly following irradiation and also levels of secreted IL-1β, IL-6, IP-10, and ISG15 were elevated.

**Conclusion:**

This study has investigated biological mechanisms in keratinocytes post-*in vitro* ionizing radiation. A common radiation signature in keratinocytes was identified. The role of IFN response in keratinocytes along with increased levels of pro-inflammatory cytokines and proteins could hint towards a possible mechanism for oral mucositis.

## Introduction

Head and neck cancer (HNC) accounted for 890,000 yearly cases and 507,000 deaths in 2017. It includes cancers in oral cavity, sinus, larynx, salivary glands, and nose (1–3). It is estimated that about 75 % of HNC patients will undergo radiotherapy treatment (RT) (4). Normal tissues, surrounding the tumour tissues, absorb ionizing radiation (IR) as an unwanted side-effect during RT. This results in acute and severe late normal tissue damage including dysgeusia, oral infections, radiation-related dental caries, and oral mucositis (OM) (5, 6). To identify at-risk patients and/or prevent severe OM, one needs to first understand the normal tissue biology post-IR or normal tissue response to irradiation.

OM is defined as the damage or inflammation that starts in the mucosal lining in the oral cavity as a result of chemo- or radiotherapy and usually occurs in the first two weeks of radiation therapy after exposure to a cumulative dose of 10-15 Gy (7). In fact, OM was reported in 80% of patients treated with RT, including 58 % with at least grade 3 mucositis (8–10). OM not only has a negative impact on quality of life (for example extreme pain and difficulty in swallowing) but is also a detrimental dose-limiting factor for RT and a reason to interrupt or even terminate radiation therapy (11). Several intrinsic and extrinsic factors, including individual radiosensitivity, determine the risk for the development of oral mucositis (12, 13).

Inflammation refers to the immune response against pathogens, damaged cells, or irritants. Interferons, cytokines, and inflammasomes involved in cytokine activation are important mediators in inflammatory processes (14). On the molecular level, a release of reactive oxygen species (ROS) and pro-inflammatory cytokines such as Interleukin 1 Beta (IL-1β) and Interleukin 6 (IL-6) that initiate inflammation is thought to be the starting point of OM (10). Signal transducer and activator of transcription (STAT)-associated proteins have been identified as key players in mediation of inflammation after IR treatment in mouse and human endothelial cells (15, 16). Within STAT protein family, STAT1 is responsible for regulating both type I and type II IFN whereas STAT2 regulates type II IFN, which is involved in the production of different pro-inflammatory and anti-inflammatory cytokines for tissue survival and homeostasis (14, 17).

In this study, we focused on keratinocytes, an epithelial cell layer, as a model for normal tissue damage following inflammation. Keratinocytes, as a first line of defence against external stressors, can provide an ideal tool for investigating acute and late effects of radiation exposure (5, 6). Several studies describe them as key players in oral mucositis (18, 19). They are both a source and target of abundant cytokines, involved in different biological processes, including cross-talk with immune cells, that influence the endpoint of mucosal diseases (20, 21). Keratinocytes also play a crucial role in the repair of epithelial wounds by interacting with fibroblasts and endothelial cells (22).

Few studies have investigated radiation effects on keratinocytes. An increase in reactive oxygen species (ROS) was reported in HaCat cells 12 h after 8 Gy X-Ray irradiation (23). iTRAQ-based proteomic analyses revealed suppression of ribosomal biogenesis in human primary keratinocytes 24 h after 0.1 Gy X-Ray irradiation (24). Besides cell death, γ-IR (0.1 Gy) was also shown to induce differentiation in Ca^2+^ -activated keratinocytes, validated by increased mRNA and protein levels of involucrin, a marker for keratinocyte differentiation (25). Biopsy-derived oral keratinocytes were recently proposed as a model to investigate individual radiosensitivity, where irradiated keratinocytes showed a dose-dependent (2 Gy-8 Gy) decrease in their spread as well as their colony forming efficiency (13).

Although there are studies for keratinocytes investigating proteins of interest after IR, no comprehensive proteomic analyses have been performed on these cells after medium to high doses of irradiation. In the present study, we used keratinocytes from micro-biopsies obtained from individuals (11). These cells have been shown to exhibit keratinocyte characteristics and provide an optimal biomaterial for examining an individual’s response to irradiation. Since the biopsies are valuable and limited material, we used a well-established keratinocyte cell line for further experiments. We investigated the late (96 h) effects of radiation exposure (6 Gy) on primary oral biopsied keratinocytes and oral keratinocyte factor 6 (OKF6) cells (26) using proteomics and validated the affected pathways in the OKF6 cell model. Data presented here will collectively enable us to understand the mechanisms behind inflammation in the oral cavity and the onset of oral mucositis following IR. Moreover, this study will be fundamental to investigate and identify proteomic signatures that might help to understand individual radiosensitivity (27).

## Materials and methods

### Oral human microbiopsy and cultivation of primary keratinocytes

Detailed information about oral micro-biopsy and the cultivation of human oral keratinocytes are provided somewhere else (13). Briefly, biopsies were performed in five individuals (University of Freiburg approval vote ETK-FR 449/16, amended by vote ETK-FR 413/17) in the vestibular mucosa tooth region (teeth 32-42). Biopsied material was stored in DMEM/F12-medium (35 mM HEPES, 100 U/mL penicillin, 100 μg/mL streptomycin, 2 µg/mL ciprofloxacin), cut into <0.5 mm pieces and keratinocytes were cultivated in Keratinocyte Growth Medium 2 (KGM 2, #C-20111, Promocell, Germany), supplemented with 5% human serum (‘off the clot’), penicillin (25 U/mL), and streptomycin (25 µg/mL).

### Cultivation of OKF6

OKF6 cell line (RRID: CVCL L222) (26) was kindly provided by Prof. Dr. Horst Zitzelsberger (Helmholtz Zentrum Munich). OKF6 growth medium consisted of 125 mL Keratinocyte SFM (Gibco-17005-034, Thermo Fisher Scientific, Illinois, USA), 125 mL 1:1 DMEM/F12 (Gibco-11330-032): F-12 Nut Mix (Gibco-21765-029, Thermo Fisher Scientific, Illinois, USA), 663 µL Bovine pituitary extract (Thermo Fisher Scientific, Illinois, USA), and 2 µL EGF (Thermo Fisher Scientific, Illinois, USA). Cells were thawed in Cell Culture Flask T-75 (Merck, Darmstadt, Germany) and washed with PBS (VWR International GmbH, Darmstadt, Germany). Cell medium was changed every three days and cells were split after 90% confluence was achieved.

### Irradiation

OKF6 cells were X-ray irradiated (XStrahl 225, West Midlands, UK) with a total dose of 6 Gy (195 kV, 0.5233 Gy/min) or sham-irradiated. Primary keratinocytes were irradiated with ^137^Cs in a Gammacell 40 Extractor (Best Theratronis, Canada) with a total dose of 6 Gy (0.67 Gy/min). IR was performed at room temperature, cells were cultivated at 37 °C for 96 h, and subsequently frozen at -80 °C.

### Protein extraction

Radioimmunoprecipitation buffer (RIPA, Thermo Fischer Scientific, Illinois, USA) containing 25 mM Tris-HCl (pH 7.6), 150 mM NaCl, 1% NP-40, 1% sodium deoxycholate, and 0.1% SDS was combined with 1X Halt™ Protease and Phosphatase Inhibitor Cocktail (Thermo Fischer Scientific, Dreieich, Germany) to lyse the cells. The frozen cell pellets were thawed on ice and incubated with lysis buffer (10 min, 72 °C). The samples were then subjected to five sonication cycles (30 sec on/30 sec off, 4 °C) on a Bioruptor® Pico sonication device (Diagenode, Liege, Belgium) followed by centrifugation (10 min, 22000 *g*, 4 °C) (28). The supernatant containing protein lysate was then transferred to new tubes. Protein concentration was determined using a colorimetric assay (RC DC™, BioRad, California, USA), as per manufacturer’s recommendation, in duplicates using bovine serum albumin (BSA) as an internal standard.

### Mass spectrometry

10 µg of OKF6 cell lysate and 5 µg of primary keratinocytes lysate were used for mass spectrometry measurements. From each sample, total proteins were proteolyzed with LysC (Wako Chemicals, Neuss, Germany) and trypsin (Promega, Mannheim, Germany), using a modified filter-aided sample preparation protocol (29, 30). Eluted peptides were analyzed on a Q Exactive HF mass spectrometer (Thermo Fisher Scientific, Waltham, MA, USA) in the data-dependent mode in a fully randomized measurement order. Approximately 0.5 µg peptides per sample were automatically loaded to the online coupled ultra-high-performance liquid chromatography (UHPLC) system (Ultimate 3000, Thermo Fisher Scientific). A nano trap column was used (300-µm ID X 5mm, packed with Acclaim PepMap100 C18, 5 µm, 100 Å; LC Packings, Sunnyvale, CA) before separation by reversed-phase chromatography (Acquity UHPLC M-Class HSS T3 Column 75 µm ID X 250 mm, 1.8 µm; Waters, Eschborn, Germany) at 40 °C. Peptides were eluted from the column at 250 nL/min using increasing acetonitrile concentration (in 0.1% formic acid) from 3% to 41% over a linear 95-min gradient. MS spectra were recorded at a resolution of 60 000 with an AGC target of 3 × 10^6^ and a maximum injection time of 50 ms from 300 to 1500 *m/z*. From the MS scan, the 10 most abundant peptide ions were selected for fragmentation via HCD with a normalized collision energy of 27, an isolation window of 1.6 *m/z*, and a dynamic exclusion of 30 s. MS/MS spectra were recorded at a resolution of 15 000 with a AGC target of 10^5^ and a maximum injection time of 50 ms. Unassigned charges, charges of +1, and above +8 were excluded from precursor selection.

### Data processing – protein identification

Proteome Discoverer 2.4 software (Thermo Fisher Scientific; version 2.4.1.15) was used for peptide and protein identification via a database search (Sequest HT search engine) against SwissProt human database (release 2020_02, 20432 sequences), considering full tryptic specificity, allowing for up to one missed tryptic cleavage site, precursor mass tolerance 10 ppm, and fragment mass tolerance of 0.02 Da. Carbamidomethylation of Cys was set as a static modification. Dynamic modifications included deamidation of Asn and Gln, oxidation of Met; and a combination of Met loss with acetylation on the protein *N*-terminus. Percolator (31) was used for validating peptide spectrum matches and peptides, accepting only the top-scoring hit for each spectrum, and satisfying the cutoff values for False Discovery Rate (FDR) <1%, and posterior error probability <0.01. The final list of proteins was complied with the strict parsimony principle.

### Data processing – label-free quantification

The quantification of proteins, after precursor recalibration, was based on abundance values (intensity) for unique peptides. Abundance values were normalized to the total peptide amount to account for sample load errors. The protein abundances were calculated by summing the abundance values for the top 3 admissible peptides. The final protein ratio was calculated using median abundance values of total replicate analyses each. The statistical significance of the ratio change was ascertained employing the approach described before (32), which is based on the presumption that we look for expression changes for proteins that are just a few in comparison to the number of total proteins being quantified. The quantification variability of the non-changing “background” proteins can be used to infer which proteins change their expression in a statistically significant manner.

Proteins identified (<1% FDR) with at least two unique peptides, in at least 50 % of the samples, and having a fold change of ± 1.5 (6 Gy/0 Gy) along with an adjusted p-value < 0.05 were considered as significantly deregulated and used for bioinformatics analyses.

### Bioinformatic analyses

*In silico* enrichment for protein-protein interactions was performed with STRING software (https://string-db.org) (33). STRING-embedded functions were used for the prediction of biological processes associated with Gene Ontology (GO) terms (34), and for mechanistic Reactome pathways (35).

### Immunoblotting

Immunoblotting was performed using Stain-Free technology (36). Briefly, 12 µg of protein lysate was denatured (10 min, 72°C) with 1× Laemmli buffer and loaded onto 4-20% Criterion™ TGX Stain-Free™ protein precast gels (Bio-Rad Laboratories GmbH, Munich, Germany). Gel electrophoresis was performed at 50 V for 15 min followed by 200 V for 1 h. The proteins were transferred into a PVDF membrane with a predefined program of 1.3 A for 7 min using Trans-Blot Turbo Transfer System (Bio-Rad Laboratories GmbH, Munich, Germany). One-two hours blocking was performed with Intercept® (TBS) Blocking Buffer (LI-COR Biosciences GmbH, Hessen, Germany). Antibodies were diluted in TBS and used according to the manufacturer’s instructions: primary antibodies were diluted 1:1000 and secondary antibodies were diluted 1:10000. MCM5 (PA5-81898), and ISG15 (PA5-31865) antibodies were purchased from Invitrogen (Invitrogen, Massachusetts, USA). MCM7 (ab2360) antibody was purchased from Abcam (Abcam PLC, Cambridge, UK) and STAT1 (sc436) was purchased from SantaCruz (SantaCruz, Biotechnology Inc, Heidelberg, Germany). From Cell Signaling (Cell Signaling Technology, Leiden, Netherlands), following antibodies were procured: MCM3 (4102S), cGAS (15102S), pSTAT1 (9177S), pSting (72650), horse radish peroxidase (HRP)-linked anti-mouse IgG (7074P2), and HRP-linked anti-rabbit IgG (707642). After the blots were incubated with primary antibodies overnight and secondary antibodies for 1 h, the blots were imaged with ChemiDoc™ (Bio-Rad Laboratories GmbH) MP imaging device. The visualization and quantification were performed with Image Lab 0.0.1 software (Bio-Rad Laboratories GmbH) where the intensities of proteins of interest were normalized against total protein loaded in the lane.

For quantification of secreted ISG15, slot-blot was performed using Bio-Dot® SF Apparatus as per the manufacturer’s instructions. Firstly, 0,45 µm Nitrocellulose membranes were first wetted with TBS buffer. Supernatant from OKF6 cell culture 96 h after 6 Gy IR was combined with PBS (60 µL media from irradiated OKF6 plus 240 µL PBS) and loaded onto the slots. Cell culture media and media of sham-irradiated OKF6 were used as controls. Vacuum was used to immobilize the proteins in the membrane and the membranes were washed with TBS. The membrane was blocked with Intercept® (PBS) Blocking Buffer for two hours, incubated with ISG15 primary antibody overnight, and finally with secondary antibody (Starbright B700 anti-rabbit, BioRad Laboratories GmbH)) for an hour. Imaging of the blots and visualization was performed as described for Immunoblotting. The intensity of ISG15 was normalized against the background using an unconditioned medium (60 µL media plus 240 µL PBS).

### Immunofluorescence microscopy

OKF6 cells were seeded in 6 cm dishes for 24 h, irradiated at 6 Gy, incubated for another 96 h, and fixed with 2% formaldehyde in Dulbecco’s PBS for 15 min. Cells were permeabilized with 0.15% Triton X-100 in PBS 3x for 5 min. Samples were blocked with blocking solution (1% BSA/0.15% glycine/PBS) and incubated with 75 µL of STAT1 (1:50, sc436, Santa Cruz Biotechnology Inc, Heidelberg, Germany), pSTAT1 (1:100, 9177S, Cell Signaling Technology, Leiden, Netherlands) and pIRF3 (1:100, 37829, Cell Signaling Technology, Leiden, Netherlands) in blocking solution overnight at 4 ^o^C in a humified chamber. After washing (5 min PBS, 10 min 0.15 % Triton X-100 in PBS, 5 min PBS and 7 min with blocking solution) slides were incubated with 75 µL of anti-rabbit IgG (H + L), F(ab’)_2_ fragment conjugated to Alexa Fluor 555 fluorescent dye (1:1000, 4413, Cell Signaling Technology, Germany) in blocking solution for 45 min at room temperature. Following 2x 5 min in 0.015% Triton X-100 in PBS and 2x 10 min in PBS, slides were mounted with 16 µL Vectashield mounting medium including 4′-6-diamidino-2-phenylindole (DAPI) (Vector Laboratories, USA). Immunofluorescence analysis and image acquisition were performed using a Zeiss AX10 microscope equipped with an Axiocam 503 mono camera. Images were processed with ZEN2.6 (blue edition) software.

### RNA isolation and reverse transcriptase polymerase chain reaction

OKF6 cells were cultivated, irradiated with 6 Gy or sham-IR treated, and frozen (-80 °C) 96 h after IR. Cell pellets were thawed on ice and RNA was isolated using RNeasy® MiniKit (Qiagen, Hilden, Germany) according to the manufacturer’s instructions. The concentration of isolated RNA was determined using Eppendorf Biospectrometer® kinetic device using 1 mm Eppendorf µ Cuvette using Nuclease-free water as blank. Absorbance at 260 nm and 280 nm were used to calculate RNA amount and protein contaminants respectively.

RNA was converted to complementary DNA (cDNA) using reverse transcription polymerase chain reaction (RT-PCR). For cDNA conversion, 370 ng RNA/sample was used and RT-PCR was performed using GoScript™ Reverse Transcription System (Promega, Walldorf, Germany), following the manufacturer’s instructions.

### Quantitative PCR

qPCR experiments were performed using iTaq™ Universal SYBR® Green Supermix (Qiagen, Hilden, Germany) as per the manufacturer’s instructions. Primers against IFNα (37) (Eurofins, Munich, Germany, custom DNA oligo forward: TCC ATG AGV TGA TBC AGC AGA, reverse: ATT TCT GCT CTG ACA ACC TCC C), IFNβ (Eurofins, Munich, Germany, custom DNA oligo forward: AAACTCATGAGCAGTGCA, reverse: AGGAGATCTTCAGTTTCGGAGG), IFNγ (Biorad, Munich, Germany, unique assay ID: qHsaCID0017614), IL-6 (Qiagen, Hilden, Germany Cat. 353458620), and OAS-2 (Qiagen, Hilden, Germany, Cat. 10025636) 96 h after IR were used. Comparative quantification method, ΔΔCt, was used to analyze relative gene expression patterns. The Ct values of a housekeeping gene, TBP1 (Qiagen, Hilden, Germany, Cat: QT00000721), were subtracted from those of target genes, resulting in ΔCt values. The ΔCt values for sham-irradiated samples were subtracted from those of irradiated samples, which provided a ΔΔCt value. Relative fold-change (n-fold) was calculated as 2^-ΔΔCt^, and averaged over four replicates to calculate the average n-fold change. A paired Student’s T-test was conducted using ΔCt values for sham- and 6 Gy irradiated samples.

### Immunoassay

The cytokines in OKF6 cell culture media were measured 96 h after 6 Gy IR using magnetic beads technology and a customized panel from Luminex™. Immunoassay was performed with ProcartaPlex™ Multiplex Immunoassay (Invitrogen, Karlsruhe, Germany) and analysed on a Bio-Plex 200 System (Bio-Rad Laboratories GmbH, Munich, Germany) according to the manufacturer’s instructions. Only cytokines yielding concentrations within the linear region of standard curves were chosen for analyses.

### Statistical software

A paired two-tailed Student`s t-test was performed for immunoblotting experiments as well as cytokine measurements and significance is reported as *p<0.05, ** p<0.01, and *** p<0.001. The statistical software R (68) was used for the PCA and Volcano plot.

## Results

### IR changes the proteome of primary human keratinocytes and OKF6 post-IR

**Figure 1:**
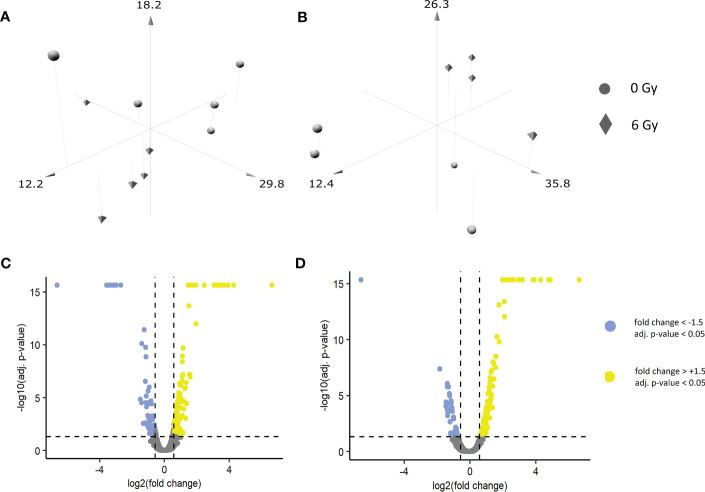
Three-dimensional PCA scatter plots of sham and irradiated samples in **(A)** primary keratinocytes and **(B)** OKF6 cells: Biopsied primary keratinocytes from five healthy volunteers as well as OKF6 cells (n=4) were *in vitro* irradiated (0 Gy, 6 Gy). Normalized abundances of the identified proteins were used to generate the plot. Volcano plot of quantified proteins in **(C)** primary keratinocytes and in **(D)** OKF 6 cells.

A shotgun-based non-targeted bottom-up approach was used to reveal the changes in the expression of proteins post-IR in biopsy-derived primary keratinocytes and in an OKF6 cell model system. In primary keratinocytes, 5879 proteins were identified, amongst which 4597 proteins were quantified (FDR <1% and identified with at least two unique peptides) (**Supplementary Material, List 1**). Similarly, in OKF6 cells, 5257 proteins were identified and 3963 proteins were quantified (**Supplementary Material, List 2**). After the primary keratinocytes were subjected to an *in vitro* 0 and 6 Gy IR, 212 proteins were significantly deregulated (fold changes of ±1.5 (6 Gy/0 Gy) and an adjusted p-value <0.05) after 96 h, which consisted of 77 downregulated and 135 upregulated proteins (**Supplementary Material, List 3**). Similarly, 169 proteins were significantly deregulated (48 downregulated, 121 upregulated) in OKF6 cells 96 h after 0 and 6 Gy IR (**Supplementary Material, List 4**). A three-dimensional principle component analyses (PCA) scatter plot (**Figures 1A, B**) revealed a separation pattern based on radiation doses (0 or 6 Gy) in both the primary keratinocytes and in OKF6 cells. Significantly deregulated proteins post-IR can be observed in volcano plots based on quantified proteins (**Figures 1C, D**).

### IFN response is predicted in the proteome of both primary human keratinocytes and in OKF6 cells post-IR

To understand the protein functions and interactions that were affected by IR, significantly deregulated proteins from both cell types were analysed for Gene Ontology (GO) terms for biological processes and Reactome pathways. The top 10 FDR-ranked GO terms for biological processes category in OKF6 cells and primary keratinocytes (**Tables 1**, **2**) revealed defense response (GO:0006852), response to stress (GO:0006950), innate immune response (GO:0045087), and interspecies interaction between organisms (GO:0044419) as shared biological processes. Similarly, IFN alpha/beta signalling (HSA-909733), IFN signalling (HSA-915351), immune system (HSA-168256), antiviral mechanism by IFN-stimulated genes (HSA-1169410), and DNA strand elongation (HSA-69190) were common in top ten ranked Reactome pathways in the two proteome profiles 96 h after exposure to 6Gy IR (**Tables 3**, **4**). Full lists of pathways are provided in **Supplementary Material (list 5-8)**.

**Table 1:** Top ten ranked gene ontology (GO) terms for affected biological processes in primary keratinocytes 96 h after 6 Gy IR.

Term ID	Term Description	Count	Strength	FDR
**GO:0006950**	**response to stress**	**78 of 3485**	**0,32**	**3,63e-07**
**GO:0006952**	**defense response**	**43 of 1296**	**0,49**	**3,63e-07**
**GO:0044419**	**interspecies interaction between organisms**	**52 of 1899**	**0,41**	**1,18e-06**
GO:0009607	response to biotic stimulus	41 of 1289	0,48	1,69e-06
**GO:0045087**	**innate immune response**	**29 of 703**	**0,59**	**2,23e-06**
GO:0051707	response to other organism	40 of 1256	0,48	2,23e-06
GO:0060337	type I interferon signaling pathway	10 of 67	1,15	1,74e-05
GO:0006955	immune response	42 of 1588	0,4	7,05e-05
GO:0009605	response to external stimulus	53 of 2310	0,33	7,05e-05
GO:0045071	negative regulation of viral genome replication	9 of 61	1,14	7,05e-05

GO terms shared with those from OKF6 cells are shown in bold. ‘Count’ represents the number of proteins annotated in this study in comparison to total proteins annotated with this term. ‘Strength’ refers to the ratio of observed enrichment effect to expected enrichment effect.

**Table 2:** Top ten ranked gene ontology (GO) terms for affected biological processes in OKF6 cells 96 h after 6 Gy IR.

Term ID	Term Description	Count	Strength	FDR
GO:0006955	immune response	59 of 1588	0,64	2,36e-18
GO:0002252	immune effector process	47 of 969	0,75	2,68e-18
**GO:0006952**	**defense response**	**53 of 1296**	**0,68**	**3,59e-18**
**GO:0006950**	**response to stress**	**83 of 3485**	**0,44**	**7,03e-17**
GO:0009615	response to virus	28 of 293	1,05	7,03e-17
GO:0002376	immune system process	69 of 2481	0,51	1,27e-16
**GO:0045087**	**innate immune response**	**38 of 703**	**0,8**	**3,52e-16**
GO:0051607	defense response to virus	24 of 210	1,12	4,73e-16
GO:0098542	defense response to other organism	42 of 900	0,74	4,73e-16
**GO:0044419**	**interspecies interaction between organisms**	**58 of 1899**	**0,55**	**4,46e-15**

GO terms shared with those from primary keratinocytes are shown in bold. Count represents the number of proteins annotated in this study in comparison to total proteins annotated with this term. ‘Strength’ refers to the ratio of observed enrichment effect to expected enrichment effect.

**Table 3:** Top ten ranked Reactome pathways in primary keratinocytes 96 h after 6 Gy IR.

Term ID	Term Description	Count	Strength	FDR
**HSA-909733**	**Interferon alpha/beta signaling**	**10 of 69**	**1,13**	**0,00018**
**HSA-913531**	**Interferon Signaling**	**14 of 196**	**0,83**	**0,00045**
**HSA-168256**	**Immune System**	**47 of 1956**	**0,35**	**0,00059**
HSA-176974	Unwinding of DNA	5 of 11	1,63	0,0019
**HSA-1169410**	**Antiviral mechanism by IFN-stimulated genes**	**9 of 81**	**1,02**	**0,0019**
**HSA-69190**	**DNA strand elongation**	**6 of 31**	**1,26**	**0,0065**
HSA-68962	Activation of the pre-replicative complex	6 of 33	1,23	0,0076
HSA-1280215	Cytokine Signaling in Immune system	22 of 681	0,48	0,0115
HSA-8983711	OAS antiviral response	4 of 9	1,62	0,0140
HSA-2559586	DNA Damage/Telomere Stress Induced Senescence	7 of 61	1,03	0,0140
HSA-140342	Apoptosis induced DNA fragmentation	4 of 13	1,46	0,0402

Pathways shared with those from OKF6 cells are shown in bold. ‘Count’ represents the number of proteins annotated in this study in comparison to total proteins annotated with this term. ‘Strength’ refers to the ratio of observed enrichment effect to expected enrichment effect.

**Table 4:** Top ten ranked Reactome pathways in OKF6 cells 96 h after 6 Gy IR.

Term ID	Term Description	Count	Strength	FDR
**HSA-913531**	**Interferon Signaling**	**26 of 196**	**1,19**	**4,86e-18**
**HSA-909733**	**Interferon alpha/beta signaling**	**18 of 69**	**1,48**	**2,19e-16**
**HSA-168256**	**Immune System**	**56 of 1956**	**0,52**	**1,53e-12**
HSA-1280215	Cytokine Signaling in Immune system	31 of 681	0,72	2,48e-10
**HSA-1169410**	**Antiviral mechanism by IFN-stimulated genes**	**12 of 81**	**1,24**	**7,78e-08**
**HSA-69190**	**DNA strand elongation**	**9 of 31**	**1,53**	**1,20e-07**
HSA-877300	Interferon gamma signaling	12 of 88	1,2	1,33e-07
HSA-176974	Unwinding of DNA	6 of 11	1,8	8,66e-06
HSA-983170	Antigen Presentation: Folding, assembly and peptide loading of class I MHC	7 of 25	1,51	1,55e-05
HSA-6798695	Neutrophil degranulation	20 of 473	0,69	1,55e-05

Pathways shared with those from primary keratinocytes are shown in bold. ‘Count’ represents the number of proteins annotated in this study in comparison to total proteins annotated with this term. ‘Strength’ refers to the ratio of observed enrichment effect to expected enrichment effect.

**Figure 2:**
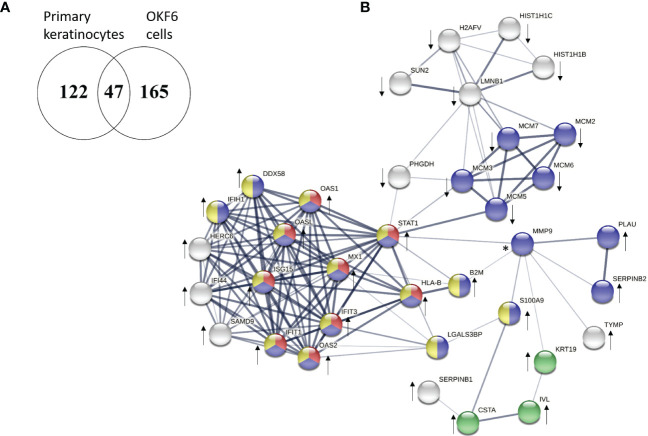
Venn diagram showing the number of deregulated proteins 96 h after 6 Gy IR-treatment in primary keratinocytes from healthy volunteers and OKF6 cells **(A)** and protein-protein interaction of the 47 common deregulated proteins between primary keratinocytes and OKF6 cells constructed with STRING (www.db-string.org) **(B)**. Colour coding shows the gene ontology (GO) terms in the category of biological processes for deregulated proteins. Red denotes type I interferon signalling pathway (GO:0060337), blue denotes response to stress (GO:0006950), yellow denotes defense response (GO:0006952), and green denotes cornification (GO:0070268). The line thickness represents the strength of data support. Proteins without an interaction partner are not shown. Proteins concordantly up- or down-regulated are denoted with arrows and proteins that are regulated in different directions are shown with an asterisk (*).

47 proteins were commonly deregulated in both keratinocytes from healthy volunteers and OKF6 cells 96 h post-IR. (**Figure 2A**; **Table 5**). Protein-protein interaction analyses performed with STRING (https://string-db.org/) revealed significant interactions among those proteins (**Figure 2B**). Proteins related to IFN responses (STAT1, MX1, ISG15, IFIT2, IFIT3, DDX58) were upregulated in both primary keratinocytes and OKF6 cells whereas proteins involved in the initiation of eukaryotic genome replication (MCM 2-7) were downregulated (**Figure 2B**). Proteins IVL and KRT19, which are associated with keratinocyte differentiation (cornification), were also upregulated 96 h after 6 Gy IR.

**Table 5:** List of common significantly deregulated proteins in primary keratinocytes and OKF6 cells 96 h after IR .

Accession	Gene symbol	Protein description	Primary keratinocytes		OKF6 cells	
			Fold change	adj p- Value	Fold change	adj p- Value
Q9BQE5	APOL2	Apolipoprotein L2	1,94	7,31E-06	2,16	2,25E-04
P61769	B2M	Beta-2-microglobulin	1,96	8,28E-05	2,43	7,93E-08
Q9NQ88	C12orf5; TIGAR	Fructose-2,6-bisphosphatase TIGAR	1,62	2,71E-03	1,68	2,21E-02
P0DP25	CALM3; CALM2; CALM1	Calmodulin-3	1,74	5,06E-05	1,61	1,98E-02
P27482	CALML3	Calmodulin-like protein 3	2,20	6,50E-08	1,97	1,26E-04
P01040	CSTA	Cystatin-A	1,53	3,75E-03	1,76	2,54E-03
O95786	DDX58	Antiviral innate immune response receptor RIG-I	1,59	6,30E-03	5,19	4,75E-16
O95864	FADS2	Acyl-CoA 6-desaturase	1,54	1,02E-02	0,53	4,42E-02
Q14161	GIT2	ARF GTPase-activating protein GIT2	2,21	2,21E-02	100	4,75E-16
P28799	GRN	Progranulin	1,97	2,98E-02	1,65	2,26E-02
Q71UI9	H2AFV	Histone H2A.V	0,642	4,47E-03	0,599	4,65E-02
Q8IVU3	HERC6	Probable E3 ubiquitin-protein ligase HERC6	1,93	3,85E-02	100	4,75E-16
P16401	HIST1H1B	Histone H1.5	0,609	5,39E-04	0,544	6,99E-03
P16403	HIST1H1C	Histone H1.2	0,492	2,40E-06	0,504	1,20E-03
P01889	HLA-B	HLA class I histocompatibility antigen, B alpha chain	2,04	1,19E-05	3,90	4,75E-16
Q8TCB0	IFI44	Interferon-induced protein 44	100	2,30E-16	100	4,75E-16
Q9BYX4	IFIH1	Interferon-induced helicase C domain-containing protein 1	1,78	2,50E-03	4,58	4,75E-16
P09914	IFIT1	Interferon-induced protein with tetratricopeptide repeats 1	3,83	2,30E-16	27,2	4,75E-16
O14879	IFIT3	Interferon-induced protein with tetratricopeptide repeats 3	2,85	2,04E-14	27,3	4,75E-16
P05161	ISG15	Ubiquitin-like protein ISG15	2,16	4,02E-09	19,7	4,75E-16
P07476	IVL	Involucrin	3,20	2,30E-16	2,14	6,86E-06
P08727	KRT19	Keratin, type I cytoskeletal 19	3,15	2,30E-16	2,33	2,62E-07
P04259	KRT6B	Keratin, type II cytoskeletal 6B	1,88	1,24E-05	1,63	1,68E-02
Q08380	LGALS3BP	Galectin-3-binding protein	1,55	1,84E-03	2,33	6,78E-07
P38571	LIPA	Lysosomal acid lipase/cholesteryl ester hydrolase	1,53	1,76E-02	1,70	2,80E-02
P20700	LMNB1	Lamin-B1	0,631	6,35E-03	0,555	1,07E-02
P49736	MCM2	DNA replication licensing factor MCM2	0,664	2,47E-02	0,497	8,36E-04
P25205	MCM3	DNA replication licensing factor MCM3	0,666	7,11E-03	0,445	2,95E-05
P33992	MCM5	DNA replication licensing factor MCM5	0,658	8,82E-03	0,423	1,02E-05
Q14566	MCM6	DNA replication licensing factor MCM6	0,664	1,13E-02	0,463	1,13E-04
P33993	MCM7	DNA replication licensing factor MCM7	0,666	1,85E-02	0,42	6,86E-06
P14780	MMP9	Matrix metalloproteinase-9	2,57	3,77E-05	0,44	2,84E-02
P20591	MX1	Interferon-induced GTP-binding protein Mx1	2,20	2,00E-10	29,4	4,75E-16
Q6ZVX7	NCCRP1	F-box only protein 50	3,87	1,01E-12	1,89	4,25E-03
P00973	OAS1	2’-5’-oligoadenylate synthase 1	2,21	2,41E-05	2,59	1,48E-06
P29728	OAS2	2’-5’-oligoadenylate synthase 2	1,51	5,54E-03	9,16	4,75E-16
Q15646	OASL	2’-5’-oligoadenylate synthase-like protein	100	2,30E-16	4,26	9,31E-13
O43175	PHGDH	D-3-phosphoglycerate dehydrogenase	0,418	3,77E-12	0,452	3,14E-05
P00749	PLAU	Urokinase-type plasminogen activator	1,50	1,86E-02	1,89	3,89E-03
P06702	S100A9	Protein S100-A9	1,57	3,05E-03	1,55	3,91E-02
Q5K651	SAMD9	Sterile alpha motif domain-containing protein 9	1,64	1,86E-03	2,50	5,23E-08
P30740	SERPINB1	Leukocyte elastase inhibitor	1,91	4,45E-07	2,07	3,48E-05
P05120	SERPINB2	Plasminogen activator inhibitor 2	2,76	2,30E-16	2,27	1,57E-06
Q01650	SLC7A5	Large neutral amino acids transporter small subunit 1	0,656	3,99E-03	0,581	2,65E-02
P42224	STAT1	Signal transducer and activator of transcription 1-alpha/beta	1,60	6,87E-04	4,03	4,75E-16
Q9UH99	SUN2	SUN domain-containing protein 2	0,599	1,23E-03	0,404	1,67E-06
P19971	TYMP; SCO2	Thymidine phosphorylase	1,82	9,12E-06	2,26	1,96E-06

### Changes in expressions of IFN-associated cytokines were observed in OKF6 cell medium post-IR

**Figure 3:**
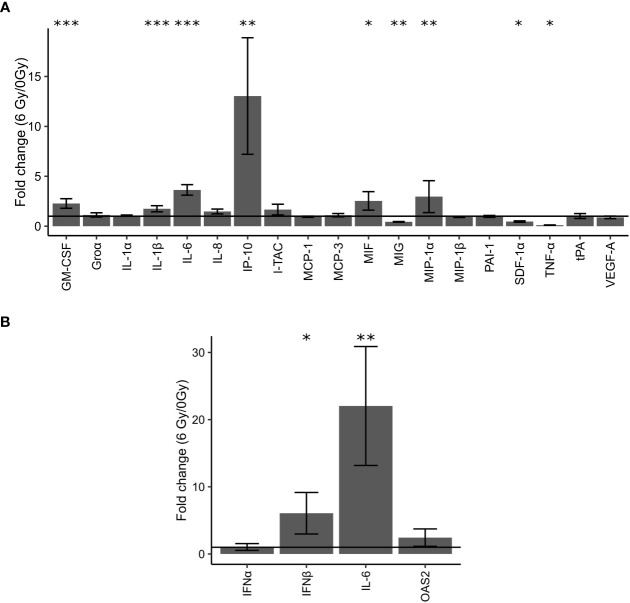
Validation of changes in protein expression in cell culture supernatants of OKF6 cells (*n* =4) 96 h after 0 and 6 Gy IR using the Bioplex system **(A)**. Fold changes in the expression of these proteins were measured: GM-CSF (Granulocyte-macrophage colony-stimulating factor), Groα (chemokine (C-X-C motif) ligand 1), IL-1α (Interleukin 1 alpha), IL-1β (Interleukin 1 beta), IL-6 (Interleukin 6), IL-8 (Interleukin 8), IP-10 (Interferon gamma-induced protein 10), I-TAC (Interferon-inducible T-cell alpha chemoattractant, MCP-1 (monocyte chemoattractant protein 1/chemokine (C-C motif) ligand 2, MCP-3 (monocyte-chemotactic protein 3), MIF (Macrophage migration inhibitory factor), MIG (Monokine induced by gamma interferon), MIP-1α (Macrophage Inflammatory Protein 1 alpha), MIP-1β (Macrophage Inflammatory Protein 1 beta), PAI-1 (Plasminogen activator inhibitor-1), SDF-1α (stromal cell-derived factor 1), TNF-α (Tumor necrosis factor alpha), tPA (Tissue plasminogen activator), and VEGF-A (Vascular Endothelial Growth Factor A). Changes in cellular mRNA levels of interferon-related genes in OKF6 cells **(B)**: Interferon alpha (IFNα), Interferon beta (IFNβ), IL-6, and OAS2(2’-5’-Oligoadenylate Synthetase 2) after 6 Gy IR **(B)**. RT-PCR was performed followed by a qPCR using TBP1 as a housekeeper gene. A line is drawn to indicate a fold change of one. *p<0.05, **p<0.01, *** p<0.001 (two-tailed Students t-test compared between 0 and 6 Gy).

To investigate a potential role of IFN response and cytokines, levels of different cytokines in the cell medium of OKF6 cell culture 96 h after 6 Gy IR were measured using the Bioplex system (**Figure 3A**; **Supplementary Materials List 9**). It was observed that GM-CSF (Granulocyte-macrophage colony-stimulating factor), IL-1β (Interleukin 1 beta), IL-6 (Interleukin 6), IP-10 (Interferon gamma-induced protein 10), MIF (Macrophage migration inhibitory factor), and MIP-1α (Macrophage Inflammatory Protein 1 alpha) were significantly upregulated whilst MIG (Monokine induced by IFNγ), SDF-1α (stromal cell-derived factor 1) and TNFα (Tumor necrosis factor alpha) were downregulated.

### Increase in cellular mRNA levels of IFNβ and IL-6 observed in OKF6 cells post-IR

Cellular mRNA levels of IFNs and IFN-related genes were measured with RT-PCR. Significant changes in mRNA levels of IFNα and OAS2 were not observed. In addition, signals for IFNγ were not detected in either irradiated or unirradiated cells. However, after IR, cellular mRNA levels of IFNβ and IL-6 significantly increased by six-fold and 22-fold after IR, respectively (**Figure 3B**).

### Proteins associated with IFN response and cellular replication were affected in OKF6 cells post-IR

**Figure 4:**
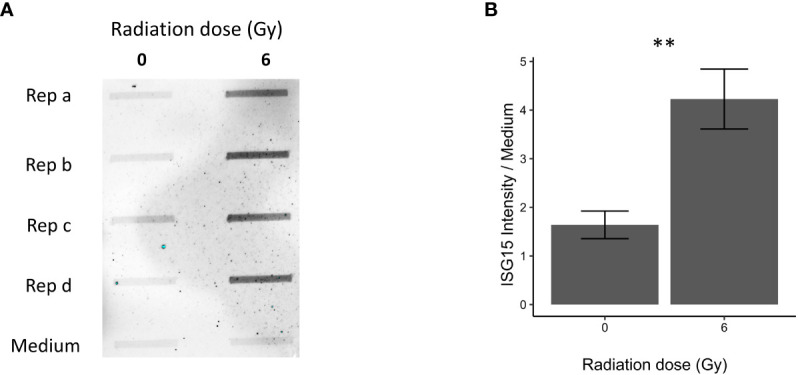
Immunoblot validation of secreted ISG15 96 h after 0 and 6 Gy IR in OKF6 cell supernatant (*n=4*). Dot blot was performed on conditioned cell culture supernatant and cell culture medium alone **(A)**. Changes in intensity of interferon-stimulated gene 15 (ISG15), normalized against medium alone, are provided in **(B)**. Error bars represent the standard deviation of four replicates and significance is reported as **p<0.01 (two-tailed Students *t*-test compared between 0 and 6 Gy).

**Figure 5:**
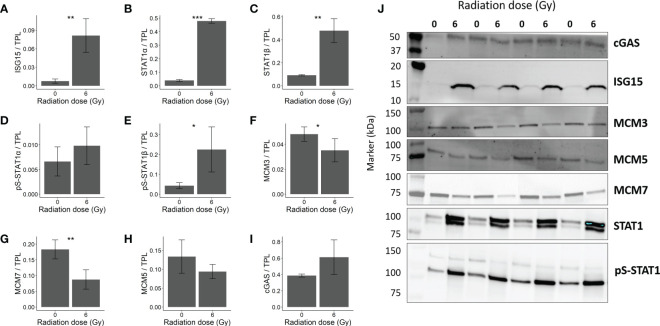
Immunoblot validation of changes in protein expression in OKF6 cells 96 h after 0 and 6 Gy IR (*n*=4). Changes in protein expression were normalized against total protein lane (TPL) using Stain-free technology. Protein expression of **(A)** ISG15 (Interferon-stimulated gene 15), **(B)** STAT1α (Signal transducer and activator of transcription 1α) (upper band), **(C)** STAT1β (Signal transducer and activator of transcription 1β) (lower band), **(D)** pS-STAT1α (phosphoserine-STAT1α), **(E)** pS-STAT1β, **(F)** MCM3 (DNA replication licensing factor MCM3), **(G)** MCM7, **(H)** MCM5, and **(I)** cGAS (cyclic GMP-AMP synthase). **(J)** Representative western blot images. Error bars represent the standard deviation of four individual measurements and significance is reported as *p<0.05, **p<0.01, and *** *p*<0.001(two tailed Students *t*-test compared between 0 and 6 Gy).

Proteins associated with IFN response and cellular replication were validated using dot blot and western blot techniques. Proteins involved in IFN response such as secreted and intracellular ISG15 were both significantly increased in OKF6 after 96 h and 6 Gy IR (**Figures 4**, **5A** respectively).

Furthermore, increased STAT1 signalling (Signal transducer and activator of transcription 1) was confirmed by elevated STAT1 alpha, beta and pS-STAT1α in the western blots (**Figures 5B, C**–**D** respectively). Also, nuclear accumulation of STAT1 and pS-STAT1 in response to irradiation hint to elevated STAT1 activity (**Supplementary Figure 3**). The increase in pS-STAT1β was not significant (**Figure 5E**). Proteins responsible for cellular replication such as MCM3 (DNA replication licensing factor MCM3) and MCM7 (DNA replication licensing factor MCM7) were downregulated (**Figures 5F, G**, respectively). However, MCM5 significant changes were not observed (**Figure 5H**). cGAS, a mediator of STING- induced IFN induction (38), showed no significant change after IR (**Figure 5I**). The blots are provided in **Figure 5J**.

## Discussion

To the authors’ best knowledge, this is the first study that reported IFN signalling in keratinocytes post-IR. Data presented here indicated that a single exposure to IR stimulates a robust type I IFN response in primary oral keratinocytes and in a keratinocyte cell culture model. Remarkably, IR induced not only an intracellular IFN signature but also increased the release of type I IFN- stimulated factors. Together these data contribute to an improved mechanistic understanding of the radiation response of keratinocytes that can be used to identify high-risk patients of RT-induced OM and/or to develop mitigation strategies for this therapy side effect in the future.

Side effects of RT on head and neck cancer (HNC) patients include oral mucositis (OM), mucosal infections, and pain as early chronic effects and tissue fibrosis; salivary gland dysfunction and sensory disorders as late tissue reactions (39). OM refers to inflammation in the mucosal lining of the oral cavity that not only affects the quality of life in patients who complain of severe pain but also is a major limiting factor for RT (8, 9, 11).

In this study, we have investigated proteomic changes in biopsied keratinocytes (13) and in oral mucosa-derived OKF6 cells 96 h after 6 Gy IR to understand biological mechanisms associated with inflammation and radiation-induced oral mucositis. Oral keratinocytes are appropriate models as they form a main barrier against external stressors, play an important role in the stimulation and maintenance of inflammatory and immunological responses, and contribute to the pathogenesis of different diseases including mucositis (5, 20, 40). A previously established model facilitated the *in vitro* propagation of keratinocytes derived from a small biopsy. Using this model, radiation effects (2 – 8 Gy) on keratinocyte migration, proliferation and cell spreading were measured (13).

In the present study, the identical cell system and previously established experimental parameters were used for the identification of IR-triggered proteomic changes and pathway regulations. Separate clustering of irradiated and sham-irradiated proteome profiles was observed in principal component analyses, suggesting marked changes induced by IR on the proteome of primary keratinocytes and OKF6 cells. Bioinformatic analysis of deregulated proteins predominantly points to immune system-related pathways, especially innate immune response and type I IFN signaling in irradiated keratinocytes. A plethora of known IFN-stimulated proteins such as HLA-protein B, IFIT1, IFIT3, IFI44, and OAS1/2 (15, 16, 41) was upregulated. Despite individual responses to IR, the increase of central type I IFN-stimulated proteins was conserved in all samples, for example, ISG15 and STAT1 (**Supplementary Figure 1**). As a second hit DNA-related processes, like strand elongation, were found. Radiation-induced proteomic signatures derived from the OKF6 model showed similarities to the primary material, further confirming the prominent role of type I IFN pathways in the radiation response of keratinocytes and also evidencing OKF6 as a reliable *in vitro* model to investigate the effect of IR. Surprisingly bioinformatic analysis of deregulated proteins did not reveal inflammasome related pathways, although the inflammasome is an important component in inflammation and in close crosstalk with interferon response pathways (14).

IFNs are cytokines traditionally associated with the immune response against foreign pathogens. They act as a first line of defence against viral infections and are responsible for immunosurveillance (42). IR-triggered type I IFN response in keratinocytes was confirmed in OKF6 cells on different levels. Firstly, type I IFN signalling was activated in OKF6 cells post-IR proven by significant upregulation of IFNβ, IL-6 and OAS2 mRNA levels. However, IFNα was not induced and type II IFNγ could not be detected. Secondly, increased levels of secreted IFN-stimulated cytokines IL-6 and IL-1β were in line with the radiation-induced type I IFN response (40, 43, 44). As potential trigger of the type I interferon response, radiation-induced phosphorylation of p-STING as well as phosphorylation and nuclear accumulation of p-IRF3 suggests the cGas-Sting pathway (45) (**Supplementary Figure 2**).

Extracellular IFNs or interleukin cytokines can bind to IFN α/β/γ receptors, and Janus kinases (JAK) are recruited to such receptors, which in turn phosphorylate STAT molecules resulting in pSTAT-dimers (42). This complex translocates to the nucleus, accumulates, and binds to chromatin resulting in pS727-STAT1 molecules, which activate its transcription activity (46, 47). However, STAT1 could also be phosphorylated by protein kinase C-delta in IFNα/β response (48). STATs are key players in IFN signalling response which mediate cellular immunity (15, 16), apoptosis (49), and differentiation (50). In primary keratinocytes, STAT1 was upregulated (fold change 1.60, adj. p-Value <0.001) but no change was observed in STAT2 levels. However, in irradiated OKF6 cells, levels of both STAT1 and STAT2 were upregulated (fold change 4.03, adj. p-Value<0.001 and fold change 2.37, adj p-Value <0.001 respectively). Moreover, a significant increase in pS727-STAT1 was observed in OKF6 cells post-IR, which indicates induction of STAT1 transcription activity.

The keratinocytes’ response to IR appears to follow similar responses to bacterial infection. Along with STAT1, MX1 and ISG15 were upregulated in the present study, similar to HUVEC cells after infection by *Rickettsia conorii* bacterium (51). ISG15 was found to be upregulated from 2-fold in primary keratinocytes up to 20-fold in OKF6 cells. Amongst hundreds of ISGs induced during IFN responses, ISG15 is one of the most strongly and rapidly induced ones (52). Intracellular ISG15 exists either as an unconjugated (free) molecule or is ubiquitin-like bound to lysine residues on target proteins (53, 54). Intracellular unconjugated ISG15 prevents degradation of USP18, a negative regulator of IFNα/β signalling and thereby stops over-amplification of IFN signals and autoinflammation (55). An increase in STAT1-associated unbound intracellular ISG15 has been previously reported in both mouse and human endothelial cells after 10 Gy IR (16, 38). Unbound ISG15 can also be secreted (56), as we found here, it was significantly increased after IR in cell culture media. This might induce IFNγ in recipient cells (57, 58), which could in turn lead to the spread of inflammation in normal tissues (15).

**Figure 6:**
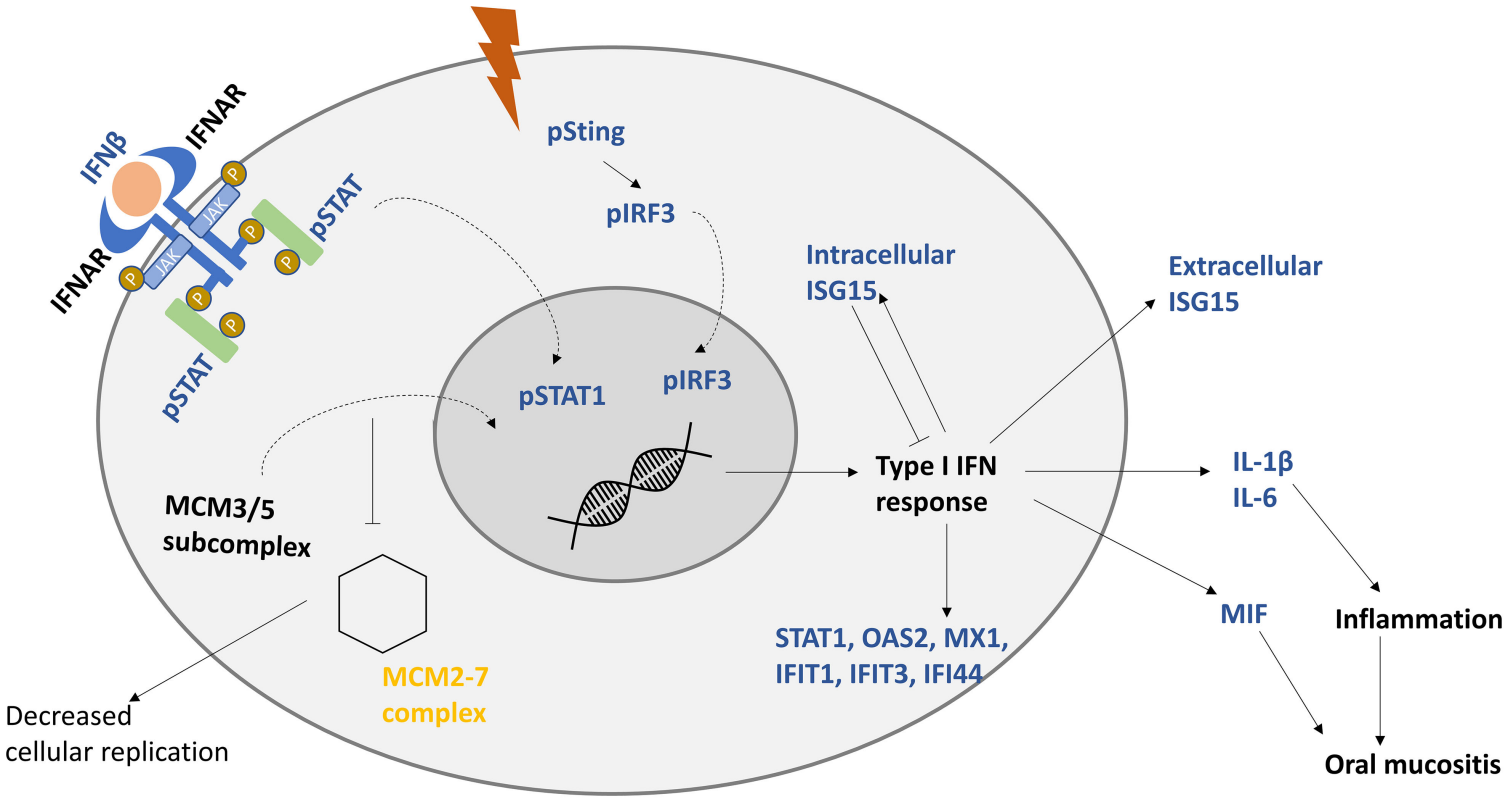
Proposed model of keratinocytes response to IR. IR induces the production of interferon beta (IFNβ), which induces type I interferon response. After interferon receptors (IFNAR) are stimulated by IFNβ, signal transducer and activator (STAT) is phosphorylated, dimerized, and translocated to the nucleus. Minichromosome maintenance protein complex 3/5 subcomplex (MCM3/5) is translocated to the nucleus, which hinders the hexamer MCM2-7 complex from being formed, decreasing cellular replication. Genes responsible for type I IFN response are activated, including interferon-stimulated gene 15 (ISG15). Intracellular ISG15 is a negative regulator of IFN response. STAT-associated production of interleukin 1 beta (IL-1β), interleukin 6 (IL-6), and macrophage migration inhibitory factor (MIF) is proposed to be responsible for inflammation, and unchecked increase in these cytokines could lead to oral mucositis. Upregulated factors are presented in blue and downregulated ones are in yellow. Curved arrows represent translocation.

Proteomics data showed the downregulation of several members of the MCM proteins including MCM 2-7. MCMs are a family of proteins that are responsible for pre-replicative complex formation, which in turn is required for DNA replication and transcription (59, 60). Cancer patients with a higher expression of MCM5 and MCM7 were significantly linked with poor overall survival (61). MCM proteins are associated with STAT-mediated response. In response to IFNγ, STAT1 was shown to recruit MCM3/5 subcomplex to the nucleus; this interaction is enhanced further by STAT phosphorylation (pS727-STAT1). By this recruitment, STAT1 was hypothesized to directly interfere with cell replication either by competition or inhibition of the MCM2-7 complex (59), as presented in **Figure 6**. This might be an explanation for why colony forming efficiency and the spread of the primary keratinocytes were decreased after IR, as described by Thomsen et al. (13), as the MCM2-7 complex is required for cellular proliferation.

IL-6, IL-1β, GM-CSF, and IP-10 were significantly upregulated in cell culture supernatants of OKF6 cells following IR (p<0.001). IL-6, a proposed biomarker for radiosensitivity (27), is one of the downstream targets of the IFN response (40, 43). It is a pro-inflammatory cytokine, which is produced in cases of infections and tissue injuries (62), including those that arise post-IR (15). IL-6 has been linked to persistent inflammation via rapid induction of acute phase proteins (63), which could lead to chronic inflammatory diseases (64). Another upregulated interleukin, which could be additionally responsible for inflammation and pain (65), is IL-1β. Both IL-6 and IL-1β were observed to be significantly higher in peri-implant mucositis compared to healthy controls (66). In contrast to IL-6 and IL-1β, anti-inflammatory effects in response to radiation exposure were reported for GM-CSF (67), and a local and systemic administration of GM-CSF showed a potential benefit in the treatment of oral mucositis (68). IP-10 was stimulated and is present for a longer time after IFNβ therapy (69) and has important roles in the attraction of immune cells and in regulation of angiogenesis during wound healing (70). As the concentrations of the three major radiation-induced cytokines are within the range of their EC50 concentrations (effective concentration required for 50% activity), there is a high probability that radiation-induced increases have potential to trigger biological effects in recipient cells.

Results of this study demonstrate that both primary keratinocytes and OKF6 cells respond to IR similar to a foreign pathogen-induced response, where STAT1-mediated IFN response is involved. In our model, IR triggered an IFNβ response leading to the production of pro- and anti-inflammatory factors which have the potential to modulate oral mucositis by deregulation of inflammatory processes. Long-term immune response due to high levels of STAT1 could perhaps be accounted for persistent inflammation. Known IFN-induced cytokines responsible for inflammation such as IL-1β and IL-6 were also found to be upregulated in the cell culture supernatants, which could help in the propagation of inflammation. MIF protein, which was reported to correlate to severe oral mucositis (71), was found to be upregulated as well.

## Conclusions

This study presents the first evidence that IR triggers an IFN response in keratinocytes, which is further mediated by STAT1. Primary keratinocytes and oral mucosa-derived OKF6 cells were used to understand normal tissue biology post-IR. Firstly with mass spectrometry, a proteomic signature of IR-response on keratinocytes was found. Based on in silico interaction analysis, candidates associated with relevant biological mechanisms, for example, immune response, interferon signalling, and DNA strand elongation were validated. Induction of IFNβ post-IR followed by the interplay of IFN-induced proteins (STAT1, ISG15) and cytokines (IL-1β, IL-6, MIF) hints towards IR-induced inflammation contributing to initiation and progression of oral mucositis that affects a majority of head and neck cancer patients. Although the cells used in our proteomic analyses were not identical, they demonstrated a very similar proteomic response to irradiation. Therefore both, the primary keratinocytes and the OKF6 cells, proved to be suitable cell models for highthroughput profiling and promised to be good biomaterials for further monitoring normal tissue reactions to radiation exposure. As a next step, the OFK6 cell model will be further explored as a tool to investigate the contribution of IFN response to the radiosensitivity of normal tissue and to assess mitigation strategies against radiation-induced inflammation. In addition, identified deregulated pathways will be studied in biopsied keratinocytes from head and neck cancer patients to identify patients at increased risk of developing oral mucositis with severe clinical manifestation (grade 3).

## Limitations of the study

Our experimental design has several limitations that may be improved in the future. First of all, keratinocytes were biopsied from five healthy individuals. A larger group is required to generalize the mechanisms triggered by ionizing radiation. Secondly, irradiation was performed *in vitro*, which does not always reflect actual biology after irradiation *in vivo*. Finally, the presented data only reflect the radiation response of keratinocytes analysed in a mono-cell culture model without considering communication with other mucosal constituents. *In vivo*, the response processes may be influenced by internal crosstalk with other mucosal components such as infiltered immune cells, fibroblasts, and other endothelial cells (72), as well as by external effects from the oral microbiome (73).

## Data availability statement

The datasets presented in this study can be found in online repositories. The names of the repository/repositories and accession number(s) can be found below: ProteomeXchange [https://proteomecentral.proteomexchange.org/], PXD040398.

## Ethics statement

The studies involving human participants were reviewed and approved by University of Freiburg approval vote ETK-FR 449/16, amended by vote ETK-FR 413/17. The patients/participants provided their written informed consent to participate in this study.

## Author contributions

Conceptualisation: MG, SH, SM, MH. Methodology: PS, KH, CS, AD, LS, FK, AT, BL, CA, UR. Investigation: PS, KH, CS, AD, LS, FK, SMH, UR. Resources: EO, AT, BL, MG, SH, SM. Formal analysis: PS, KH, AD, OA, SMH, SH. Writing-original draft preparation: PS, LD. Writing- reviewing and editing: AD, SM, OA, UR, MG, SMH, SH. Supervision: SM, OA, MG, UR, MH. Project administration: MG, SH, MH, SM. Funding acquisition: MH, MG, SH. All authors contributed to the article and approved the submitted version.

## Supplementary material

Supplementary Figure 1 Individual responses to ionizing radiation: Fold changes of STAT1, ISG15, MCM3, and MCM7 in primary keratinocytes biopsied from five different volunteers based on normalized abundances obtained from LC-MS^n^ analyses. Primary keratinocytes were 6 Gy or 0 Gy *in vitro* irradiated and cells were lysed 96 h after irradiation.

Supplementary Figure 2 Phosphorylation of STING in OKF6 cells (n=4) 96 h after 6 Gy IR (A). Fold change of p-STING (Ser366) in total protein lysates (B). Phosphorylation and nuclear localisation of IRF3 in OKF6 cells 96 h after 6 Gy IR detected by immunofluorescence microscopy (B), p-IRF3(Ser396) is shown in orange, nuclei are shown in blue. A representative image is shown.

Supplementary Figure 3 Phosphorylation and localization of pS-STAT1 and STAT1 in OKF6 cells 96 h after 6 Gy detected by immunofluorescence microscopy. pS727-STAT1 (A) and STAT1 (B) are shown in orange, nuclei are shown in blue. A representative image is shown.

Supplementary Figure 4 Total protein amounts loaded on membranes for protein quantification in immunoblot experiments. Proteins detected are shown above the membrane images.
