# Supplementary figures and images for: Towards unravelling biological mechanisms behind radiation-induced oral mucositis via mass spectrometry-based proteomics
Source: Front Oncol. 2023 Jun 13;13:1180642. doi: 10.3389/fonc.2023.1180642 (PMC10298177; doi:10.3389/fonc.2023.1180642)

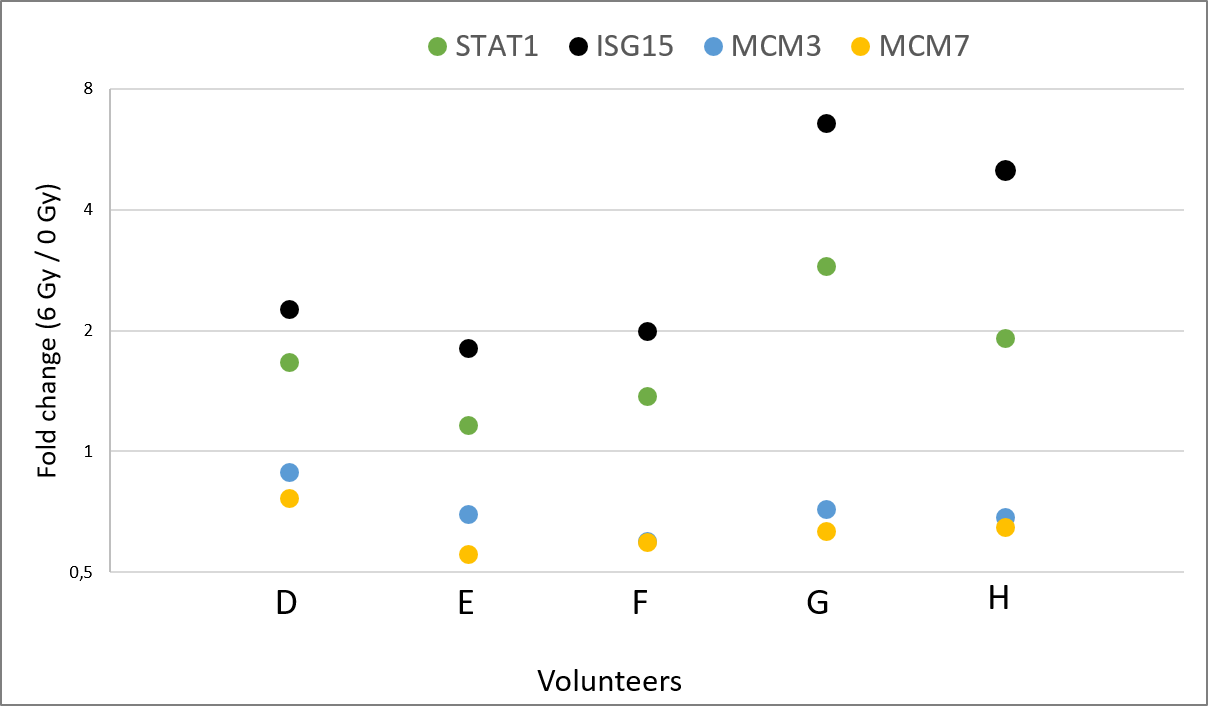

Supplement: Supplementary Figure 1 — Individual responses to ionizing radiation: Fold changes of STAT1, ISG15, MCM3, and MCM7 in primary keratinocytes biopsied from five different volunteers based on normalized abundances obtained from LC-MSn analyses. Primary keratinocytes were 6 Gy or 0 Gy in vitro irradiated and cells were lysed 96 h after irradiation. [file Image_1.tif]

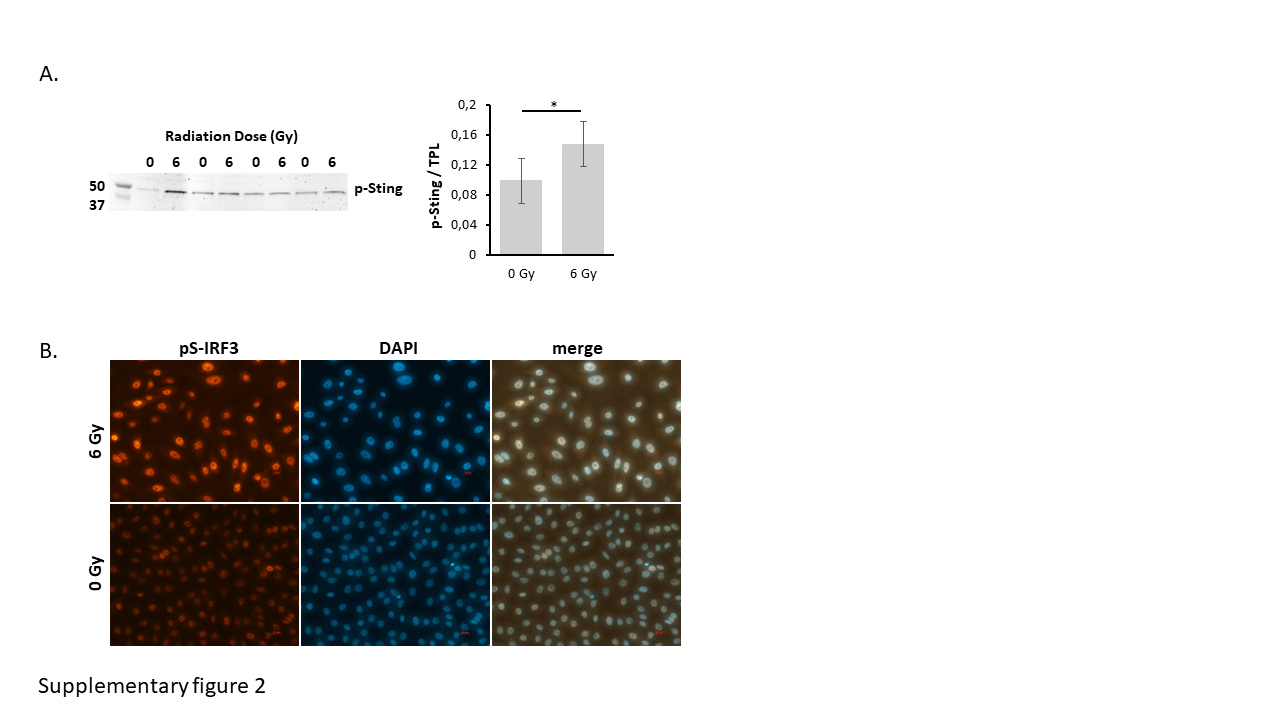

Supplement: Supplementary Figure 2 — Phosphorylation of STING in OKF6 cells (n=4) 96 h after 6 Gy IR (A). Fold change of p-STING (Ser366) in total protein lysates (B). Phosphorylation and nuclear localisation of IRF3 in OKF6 cells 96 h after 6 Gy IR detected by immunofluorescence microscopy (B), p-IRF3(Ser396) is shown in orange, nuclei are shown in blue. A representative image is shown. [file Image_2.tif]

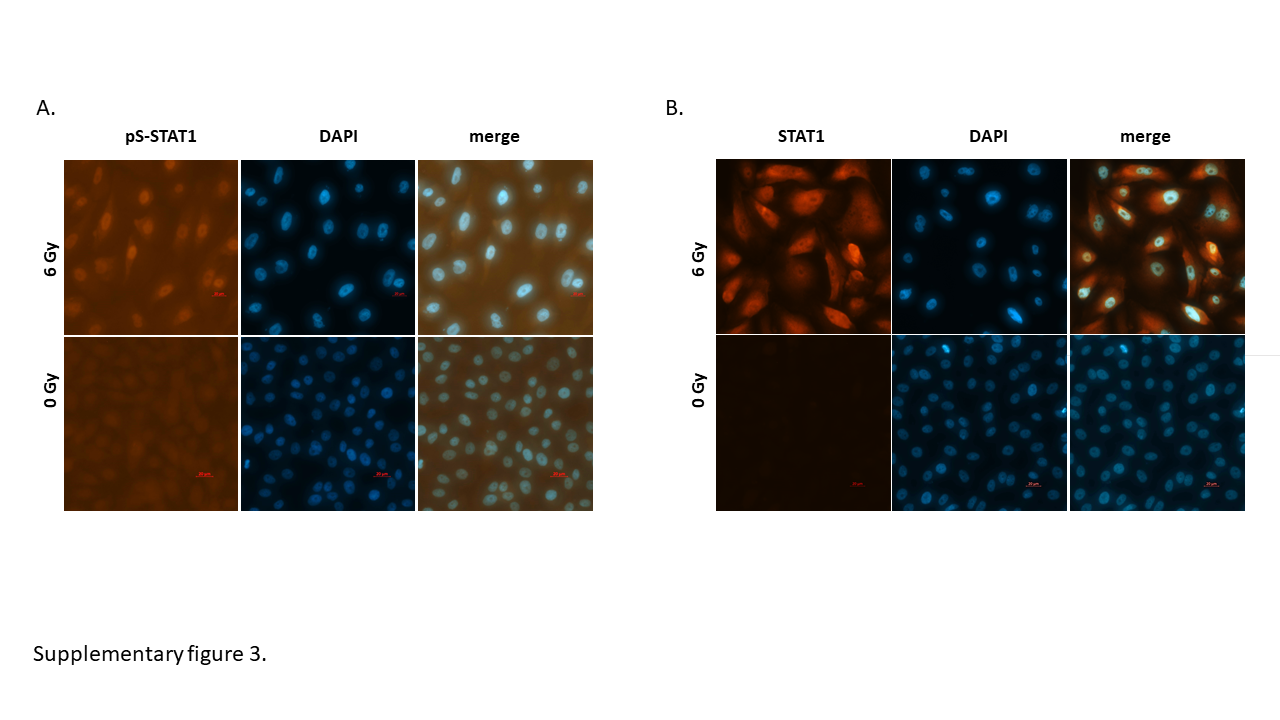

Supplement: Supplementary Figure 3 — Phosphorylation and localization of pS-STAT1 and STAT1 in OKF6 cells 96 h after 6 Gy detected by immunofluorescence microscopy. pS727-STAT1 (A) and STAT1 (B) are shown in orange, nuclei are shown in blue. A representative image is shown. [file Image_3.tif]

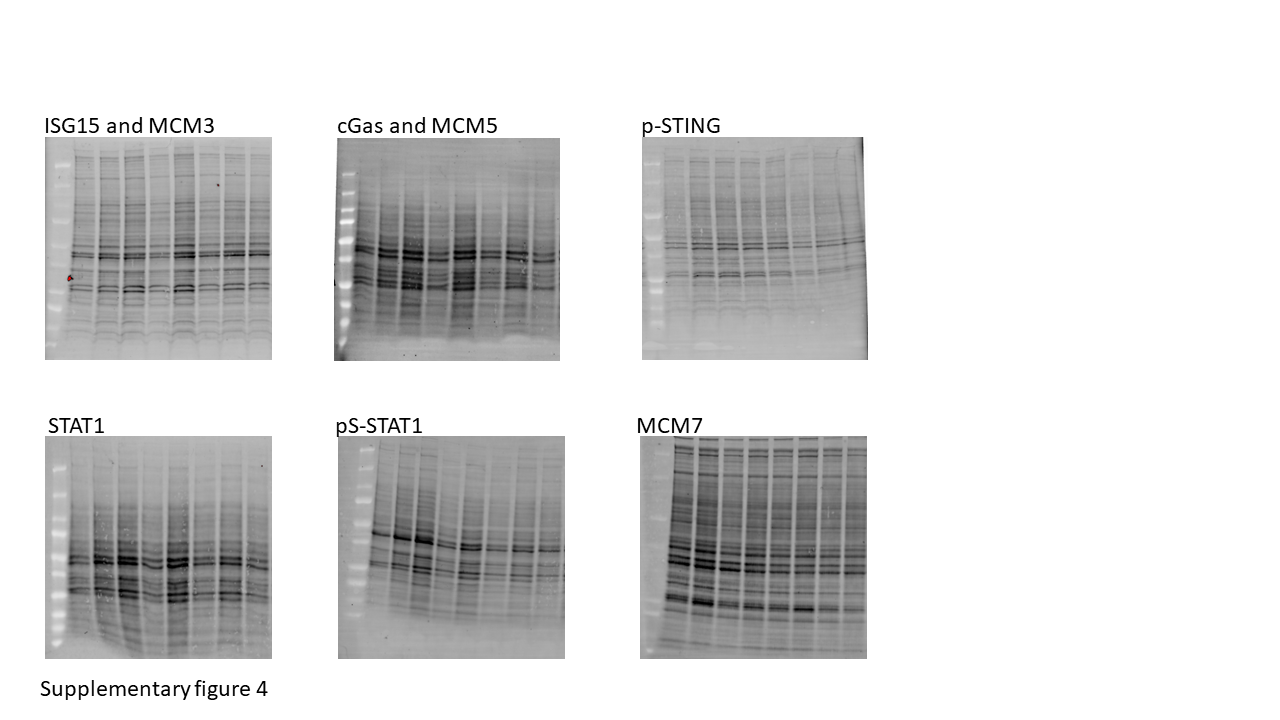

Supplement: Supplementary Figure 4 — Total protein amounts loaded on membranes for protein quantification in immunoblot experiments. Proteins detected are shown above the membrane images. [file Image_4.tif]
